# An engineered thermally tolerant apo-cytochrome scaffold for metal-less incorporation of heme derivative

**DOI:** 10.1371/journal.pone.0293972

**Published:** 2023-11-09

**Authors:** Sami Ben Aoun, Sheikh Muhammad Ibrahim

**Affiliations:** 1 Faculty of Science, Department of Chemistry, Taibah University, Al-Madinah Al-Munawarah, Saudi Arabia; 2 Faculty of Science, Chemistry Department, Islamic University of Madinah, Al-Madinah Al-Munawarah, Saudi Arabia; Shiv Nadar University, INDIA

## Abstract

Cytochrome *c*_552_ from *Thermus thermophilus* is one of the hot topics for creating smart biomaterials as it possesses remarkable stability, is tolerant to multiple mutations and has therefore been recently reported for a number of functionalizations upon substitution of the original prosthetic group with an artificial prosthetic group. However, all of the substitutions were driven by the coordination through the axial ligands followed by complete reconstitution with a metal–porphyrin complex. This limits the scope of the cytochrome c for incorporating a metal-less non-natural heme species that could improve the versatility of cytochrome c for a new generation of engineered cytochrome proteins for further enhancement in their functionalities such as biocatalysts. In this connection, a new variant of Cytochrome c (*rC*_552_ C14A) from *Thermus thermophilus* was reported, where an easy approach to remove the original prosthetic group was achieved, followed by the incorporation of a number of metal-PPIX derivatives that ultimately led to the formation of artificial c-type cytochromes through covalent bonding. The apo-cytochrome was found to be thermally tolerant and to possess a distinctive overall structure as that of the wild type, as was evident from the corresponding CD spectra, which ultimately encouraged reconstitution with a metal-less protoporphyrin derivative for better understanding the role of axial ligands in the reconstitution process. Successful reconstitution was achieved, resulting in a new type of Cytochrome *b*-type artificial protein without the metal in its active site, indicating the non-involvement of the axial ligand. In order to prove the non-involvement of the axial ligand, a subsequent double mutant (C14A/M69A) was constructed, replacing the methionine at 69 position with non-coordinating alanine residue. Accordingly, the apo-C14A/M69A was prepared and found to be extremely stable as the earlier mutants and the WT showed no signs of denaturation, even at the elevated temperature of 98°C. Subsequently, heme *b* was successfully incorporated into the apo-C14A/M69A, which demonstrated itself as a highly thermally tolerant protein scaffold for incorporating a metal-less artificial prosthetic group in the absence of the axial ligand. Further improvement in the reconstitution process is achieved by replacing the methionine at 69 position with phenyl alanine (C14A/M69F mutant), resulting in further stabilization of heme species, possibly through non-covalent π–interactions, as corroborated by molecular docking.

## 1. Introduction

Artificial metalloenzymes based on hemoproteins have gained considerable attention in the last few years as they show a promising new generation biocatalyst possessing specific catalytic activity with high efficiency [1–3]. One of the reasons behind choosing the hemoproteins for such biocatalysts is that they demonstrate a wide range of diverse but distinctive functions in the biological system [4–7]. A number of achievements were accomplished especially with well-known hemoproteins such as Hemoglobin and Myoglobin as it is easy to exchange the original prosthetic heme group with an artificial cofactor [3,8–10]. However, a recent trend shows a shift to utilizing more specific cytochrome scaffolds from more general hemoproteins towards the foundation of such artificial enzymes [11–14]. One of the ideas behind this shift is the electron transfer property of cytochrome proteins that induce an additional advantage towards the construction of a biocatalyst [15–17].

Cytochrome c is unique among all Cytochrome proteins as is carries a distinctive covalent linkage with the heme and shows a high thermal stability [18–20]. Efforts have been made for more than a decade to substitute the original heme group through reconstitution with an aritifical metallo-porphyrin as it is more challenging to exchange a natural heme c. A number of artificial cofactors such as alternative metal-substituted porphyrin rings are studied and reported in great detail in this connection [21,22]. Attempts have also been made to reconstitute with artificial non-porphyrin metal complexes and artificial metalloporphyrinoids to yield biohybrid catalysts [8,9,23]. However, all of these reconstitutions are highly restricted to metal complexes as they are driven through axial ligand coordination with the metal in the cytochrome scaffold [8]. In spite of these, axial ligand-less cytochrome proteins may be promising candidates for the future generation of biocatalysts, as is evident from some reports [24]. A recent breakthrough was achieved in this regard when an artificial peroxidase was designed through the exchange of axial ligand in Cytochrome c [25–27]. However, those reports lack durability and thermal stability and suffer from heme degradation, limiting its widespread application. In addition to this, facile conversion of the original prosthetic group with an artificial one for these types of cytochrome c is also challenging. Furthermore, porphyrin-based photosensitizers have also been indicated to be precocious candidates for photo-dynamic therapy in recent years, and attempts have been made to design more effective and ideal candidates for the same [28–31]. In recent years, the immobilization of those artificially designed prosthetic groups has also been introduced inside the protein scaffold to further innovate advanced photosensitizers [32,33]. Therefore, a highly thermostable cytochrome c scaffold, where easy and efficient substitution of the original prosthetic group with an artificially designed prosthetic group could be achieved in the absence of the axial ligand, is more desirable and could be a game-changer in the field.

**Fig 1:**
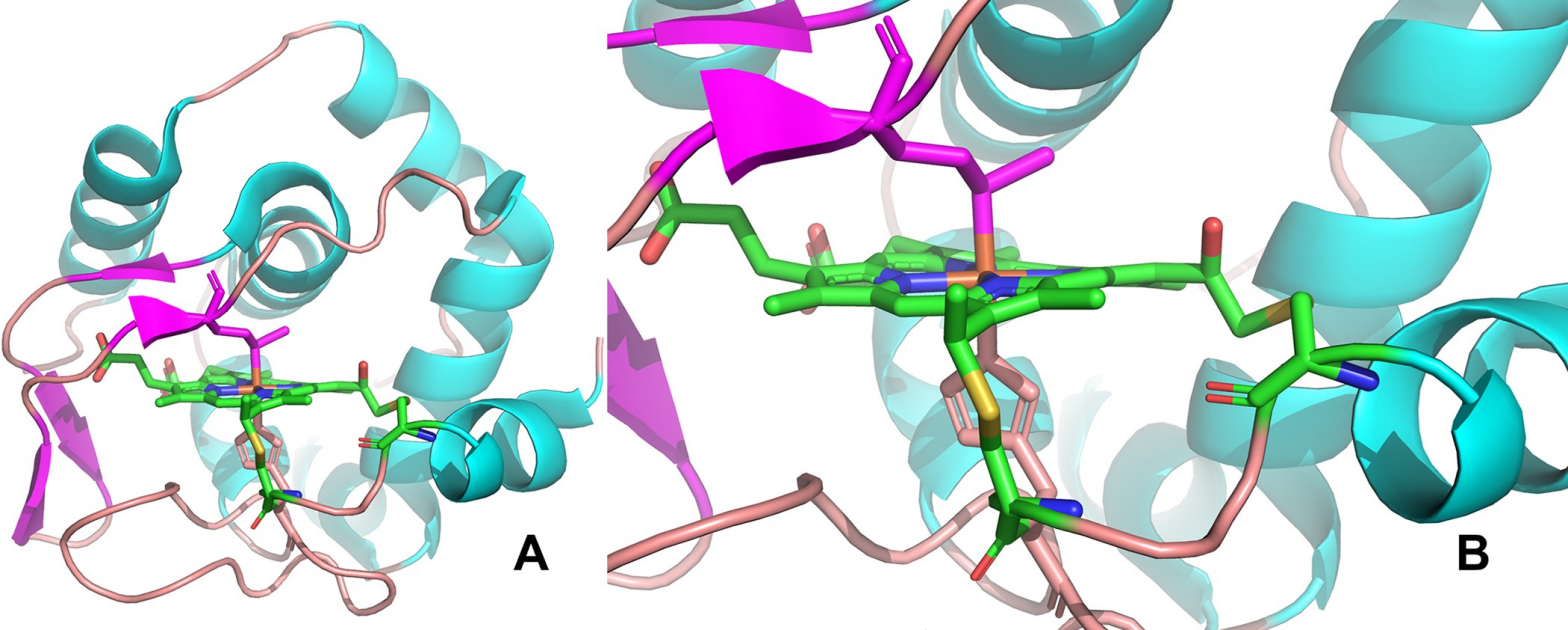
Crystal structure of thermally tolerant Cytochrome *c*_552_ WT from *thermus thermophilus* (PDB Entry: 1C52). **(**A) Whole protein and (B**)** active site. The natural heme moiety is covalently connected with the Cys11 as well as Cys14 residues and the native Fe ion is axially coordinated through Met69 and His15 residues, where Met69 is present on the distant side.

Cytochrome *c*_552_ from an extremely thermophilic bacteria, *Thermus thermophilus*, is widely investigated for its distinctive structure and thermally tolerant behavior (Fig 1) [34,35]. It was subsequently expressed in *E*. *Coli* through a recombinant technique and purified for further investigation into, e.g., the protein folding dynamics [36,37]. In addition to this, the role of the axial ligand was also well-studied in terms of folding dynamics [38,39]. All of these reports firmly established Cytochrome *c*_552_ from *Thermus thermophilus* as a potent scaffold for creating thermally tolerant biocatalysts by fulfilling its criteria to reach extremely high stability.

Recently, we achieved the engineering of the Cytochrome *c*_552_ from *Thermus thermophilus* through biogenesis and a chemical process (*rC*_552_ C14A) where we established a facile exchange process of the native heme species with artificial metal-protoporphyrin rings with the retention of its folding structure in apo-form as well as in the obtained reconstituted Cytochrome proteins [40]. We also showed a technique to create artificial c-type cytochrome proteins based on the apo-Cyt protein through a covalent linkage formation between the vinyl group of incorporated external metalloporphyrins and the free Cysteine 11 residue in the apo-Cyt scaffold with mechanistic details [41]. Further analysis of the apo-Cyt protein showed its capabilities to maintain its high thermal stability with the retention of a distinctive structure, which encouraged us to perform further reconstitution with simple protoporphyrin without the metal to check the role of axial ligand to incorporate artificial heme species. To our surprise, the apo-Cyt was able to incorporate the metal-less simple porphyrin, albeit a slow process, which suggested that the axial ligand is not essential in the reconstitution process. In order to establish this fact, we subsequently prepared an engineered double mutant, C14A/M69A, where the axial ligand was replaced with a non-coordinating alanine group, and obtained the apo-C14A/M69A through the facile removal process established earlier. According to our prediction, the apo-C14A/M69A mutant is likewise found to be stable and is able to incorporate metal-protoporphyrins in the absence of the sixth ligand. However, even though the double mutant in its apo- and reconstituted forms is found to be stable under normal conditions, the thermal stability is found to be somewhat compromised at elevated temperatures in the presence of a denaturing agent when compared with that of the wild-type (WT) Cytochrome c. To overcome this problem, a new mutant, C14A/M69F, was prepared, rationalizing that the phenyl alanine would further stabilize the incorporated protoporphyrins (PPIX) in the scaffold through π–interactions. In accordance with our belief, the new C14A/M69F double mutant was found to be extremely stable in its apo-form as well as upon incorporation of artificial metalloporphyrins and did not pass through heme degradation even after prolonged irradiation of strong light and passing through strong hydrophobic columns. Additionally, temperature-dependent CD spectra were carried out, which established its very high thermal stability in its apo-form as well as in the reconstituted protein. This is in accordance with earlier reports wherein it was shown that the removal of axial ligand in the WT does not significantly alter its thermal behavior [39]. In summary, two new double mutants were constructed, i.e., C14A/M69A and C14A/M69F, and subsequently purified in the apo-form. The aim of the former was to remove the axial ligand on the distant side through the substitution of the methionine with alanine residue that were no longer able to make an axial bond. In the latter, a phenylalanine residue containing an aromatic group was introduced, expecting an interaction with pyrrole aromatic rings of incorporated heme species. As expected, both mutants were able to reconstitute with heme *b* and metal-free porphyrin rings, respectively, through spectrometric titrations, virtually eliminating the possibility of any axial ligand interactions as it no longer contained a metal ion for making an axial bond. CD spectra were carried out before and after the reconstitution to prove their high thermal stability, particularly for the C14A/M69F mutant. The unusual thermal stability of C14A/M69F was explained by molecular docking simulation, which shows various interactions with incorporated metal-free porphyrin and different amino acid residues in the engineered protein scaffold after the reconstitution, stabilizing it even in the absence of the axial ligands. Therefore, the present work describes the first successful attempt to incorporate a metal-less protoporphyrin into a thermally tolerant Cytochrome scaffold. This result diminishes the requirement of a metal in the prosthetic group for the reconstitution process and indicates the non-necessary involvement of an axial ligand in the reconstitution process. Additionally, the vacant site created by the removal of the axial ligand provides room for a substrate binding site upon reconstitution of other metal-less non-natural compounds and chromophores into the apo-Cytochrome scaffold to obtain a new generation of engineered proteins for futuristic applications.

## 2. Results

### 2.1. Reconstitution of apo-C14A with simple porphyrin without the metal

**Fig 2:**
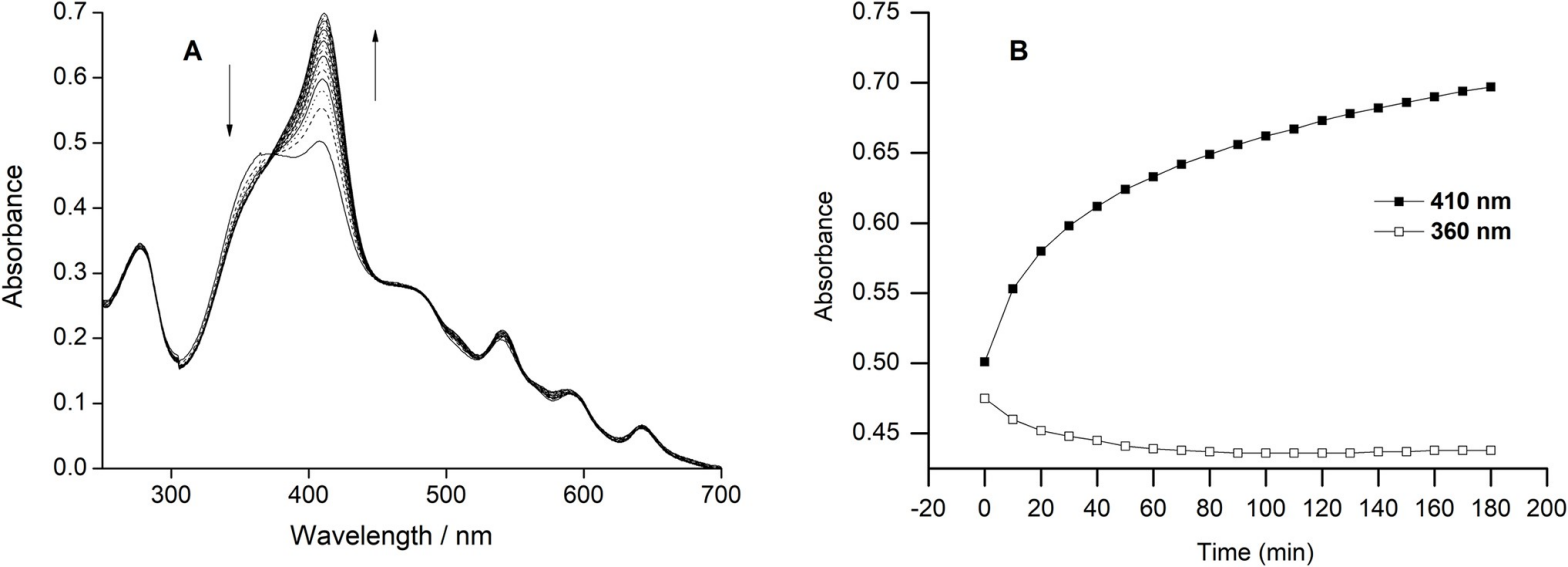
Time-dependent response of apo-C14A with the simple-protoporphyrin IX without metal. (A) 5 μM of apo-protein was mixed with one equivalent of metal-free porphyrin. The arrows indicate the direction of change in absorbance intensity with interval time of 10 minutes. (B) The increase in the absorbance at 410 nm (thick line) and decrease in the absorbance at 360 nm (thin line) are shown in every 10 minutes interval time.

Reconstitution of apo-C14A was performed with *b*-type heme without the metal in the center (metal-free porphyrin), and it shows an incorporation of simple porphyrin inside the apo-scaffold, although was not completed even after more than two hours of mixing time. Time-dependent UV–Vis spectra show a rather slow reconstitution process while mixing equal concentrations of apo-C14A with PPIX (Fig 2), unlike the previous reports where reconstitution was achieved instantaneously with metalloporphyrins [40,41]. The saturation in the 360 nm band is achieved within an hour indicating the heme is removed from the solution, whereas the no saturation was achieved for the Soret peak at the 410 nm band indicating the incorporation was not quantitative to obtain a complete recovery of the holo-protein.

### 2.2. Preparation of apo-C14A/M69A mutant

**Fig 3:**
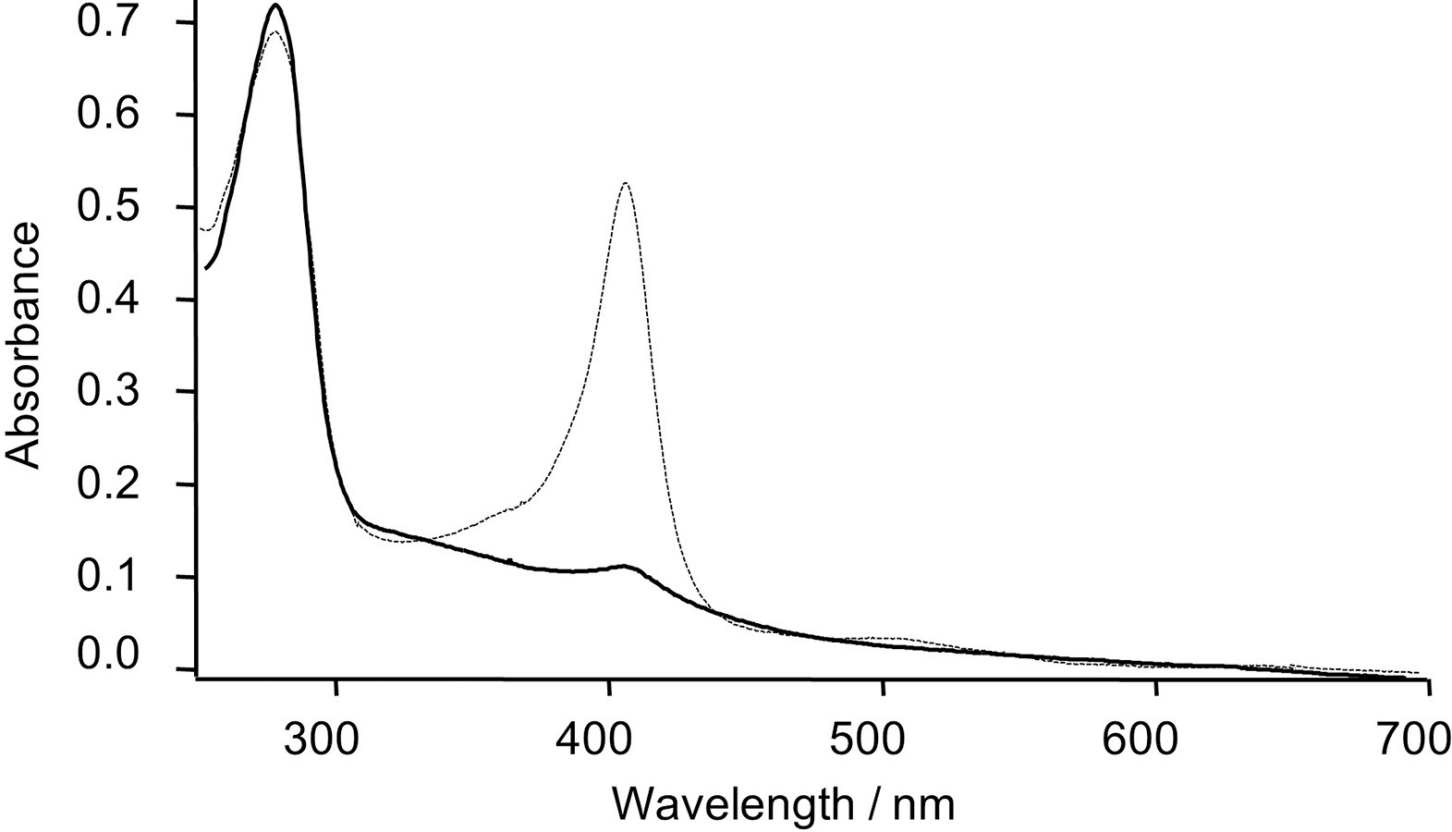
The UV–Vis spectra of C14A/M69A mutant. Immediately after the purification (dotted line) before the butyl-sepharose column treatment; after the butyl-sepharose column operation (solid line).

The engineered C14A/M69A double mutant was prepared from the C14A through the replacement of Met69 with alanine (M69A) to evaluate the role of the sixth ligand to incorporate the heme species in its scaffold. Although the SDS-PAGE gel analysis showed a single band assignable to C14A/M69A after the purification, the intensity ratio of the bands in the UV–Vis spectrum (Soret over 280 nm) was 0.77 (Fig 3). This was significantly low compared with our earlier C14A mutant which was found to be 3.48 [40]. This finding indicates that the apo-form is a major product in the purified C14A/M69A with a retention of 80% apo-form. This is unlike the case of a previous report wherein the removal of the axial ligand resulted in complete recovery of apo-protein in the absence of holoprotein, indicating a more stable nature of the engineered double mutant in the present scenario [42,43]. The Soret band of C14A/M69A was observed at 402 nm assignable to a 5-coordinated heme species due to the removal of the axial ligand Met69. A residual amount of holo-form was quantitatively removed from the apo-C14A/M69A by the butyl-Sepharose column (Fig 3).

### 2.3. Reconstitution of apo-C14A/M69A mutant

**Fig 4:**
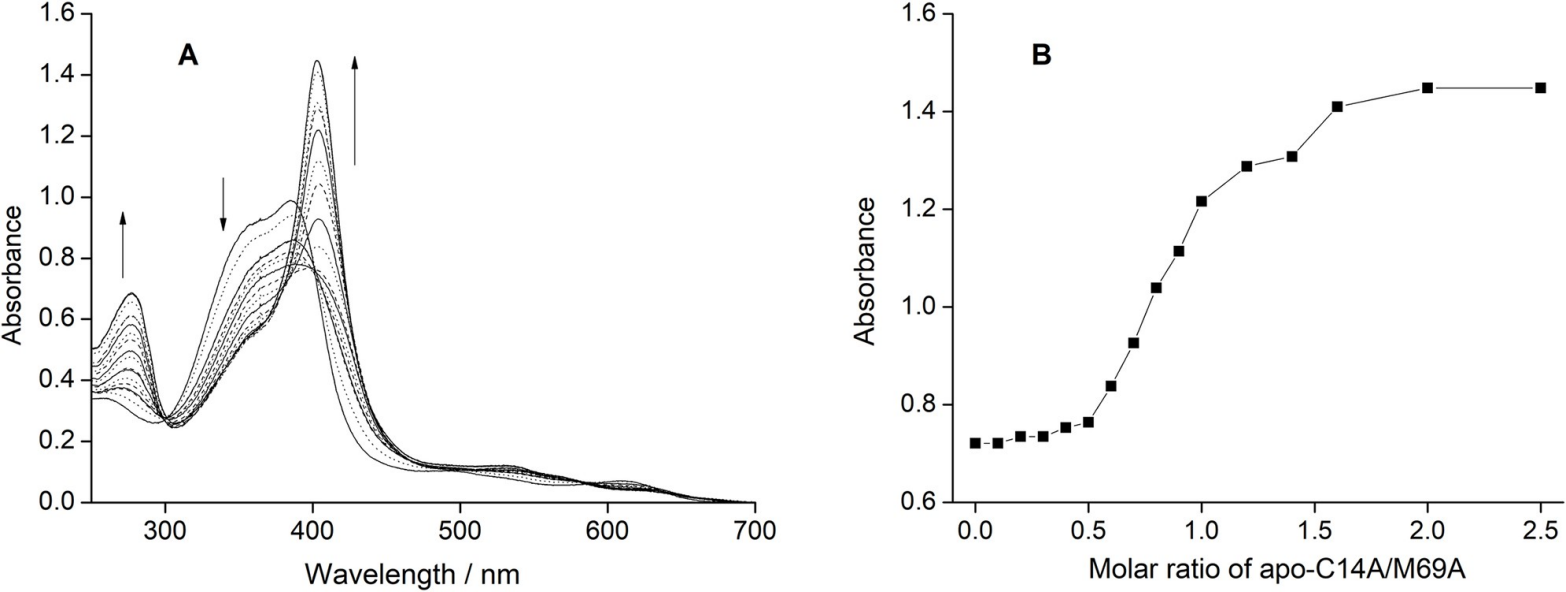
The spectrophotometric titration of apo-C14A/M69A with the ferric heme *b*. (A) The apo-protein is reconstituted with the ferric heme *b* to yield a corresponding 5-coordinated heme species. The arrows indicate the direction of change in absorbance intensity with the addition of ferric heme-*b*. (B) The absorbance at 410 nm is plotted against Molar ratio of [heme]/[apo-C14AM69A], showing a saturation of the Soret band.

The titration of apo-C14A/M69A to the ferric heme*b* (10 μM) at 25°C and pH 5.0 is shown in Fig 4. Bands characteristic of the ferric heme*b* were replaced with those assignable to a 5-coordinated ferric cytochrome b (determined from the appearance of the Soret band at 402 nm) [44,45]. It required 5 minutes to reach equilibrium after each titration of apo-C14A/M69A. This is much slower than that observed for the previous C14A mutant with ferric heme*b* [40]. No further change was observed after titration of a stoichiometric amount of apo-C14A/M69A to the heme *b*. No change was observed in the final visible spectra of the reconstituted C14A/M69A before and after the desaltation column operation, repeated dialyses, or passing through an anionic exchange column, indicating tight binding of hemin to apo-C14A/M69A mutant even in the absence of a sixth ligand.

### 2.4. Thermally tolerant behavior of apo-C14A/M69A

The expression of the engineered C14A/M69A double mutant resulted in a mixture of apo- and holoproteins which could not be separated by SEC column, suggesting a comparable volume for apo- and holoprotein. In addition to this, the CD spectrum of the engineered apo-C14A/M69A double mutant suggested that it retained its secondary structure as that of the *rC*_552_ C14A mutant reported earlier as well as the WT (cf. S1 Fig in S1 File). This concluded that the mutation in the axial ligand at 69 position does not affect the conformation of the protein and its folding structure. A temperature-dependent CD spectrum of apo-C14A/M69A was performed in succinic acid buffer, where no apparent denaturation was observed until a temperature as high as 95°C. However, in the presence of 2 M guanidine·HCl, the melting point of the apo-C14A/M69A sharply decreased to around 46.5°C, which is calculated by identifying the midpoint of CD spectra where the folded and unfolded protein structures are present in equal fractions (cf. S2 Fig in S1 File).

### 2.5. Preparation of apo-C14A/M69F mutant

Similar to the case of engineered C14A/M59A mutant, the C14A/M69F double mutant was prepared starting with C14A mutant by replacing Met69 with phenylalanine (M69F). A similar result was observed upon the purification in which a mixture of apo- and holo-form was obtained, where the holo-form appeared to form a five-coordinated heme species. A residual amount of holo-form was removed by passing the mixture through butyl-Sepharose column quantitatively as previously mentioned.

### 2.6. Reconstitution of apo-C14A/M69F mutant

**Fig 5:**
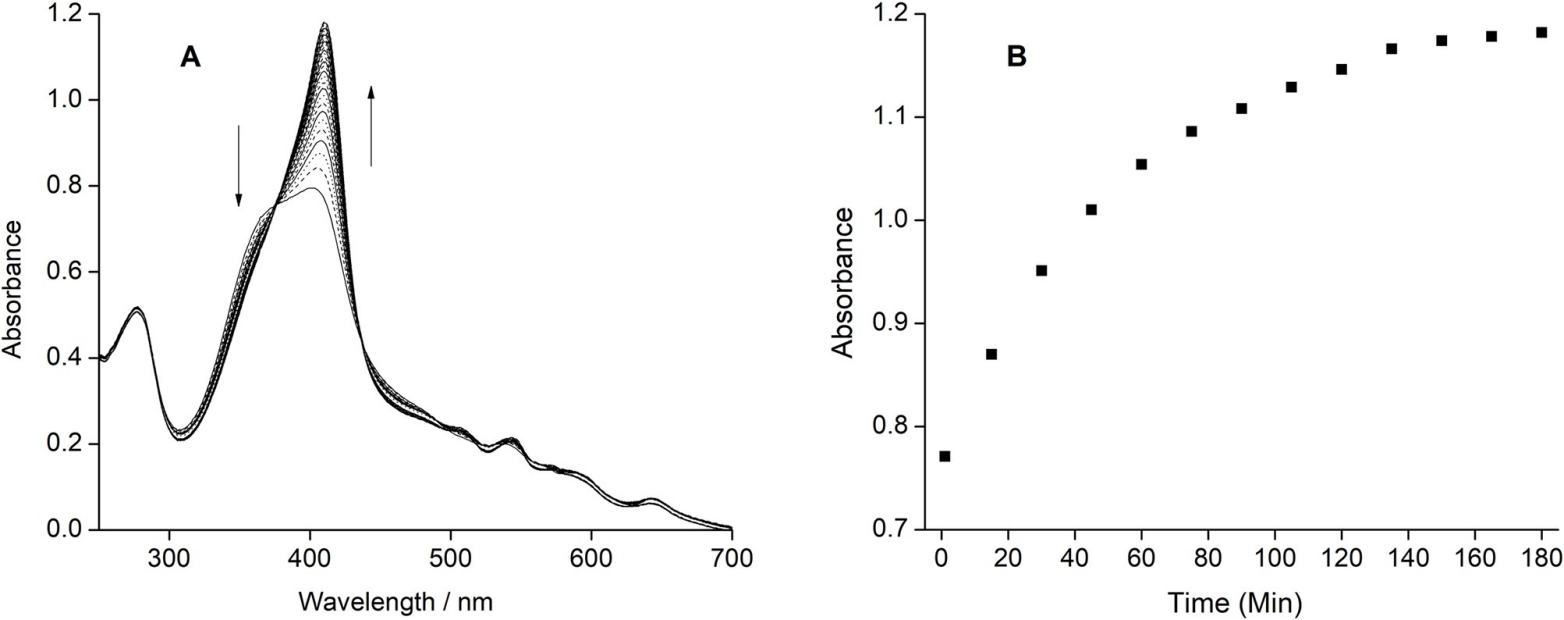
The time-dependent response of apo-C14A/F69F with the metal-less simple-PPIX. (A) 5 μM of apo-protein was mixed with one equivalent simple PPIX. The arrows indicate the direction of change in absorbance intensity with an interval time of every 15 mins. (B) The increase in the absorbance at 410 nm.

Reconstitution of apo-C14A/M69F was performed with metal-free porphyrin, which resulted in quantitative binding where the complete incorporation was achieved in 2 hours, which is rather slow compared with the C14A or C14A/M69A double mutant, suggesting that the phenyl group resulted in steric hindrance to accommodate the porphyrin ring (Fig 5). Near saturation of the Soret band at 410 nm appeared within 2 hours of the mixing time, indicating more stabilization in the heme site after the reconstitution.

**Fig 6:**
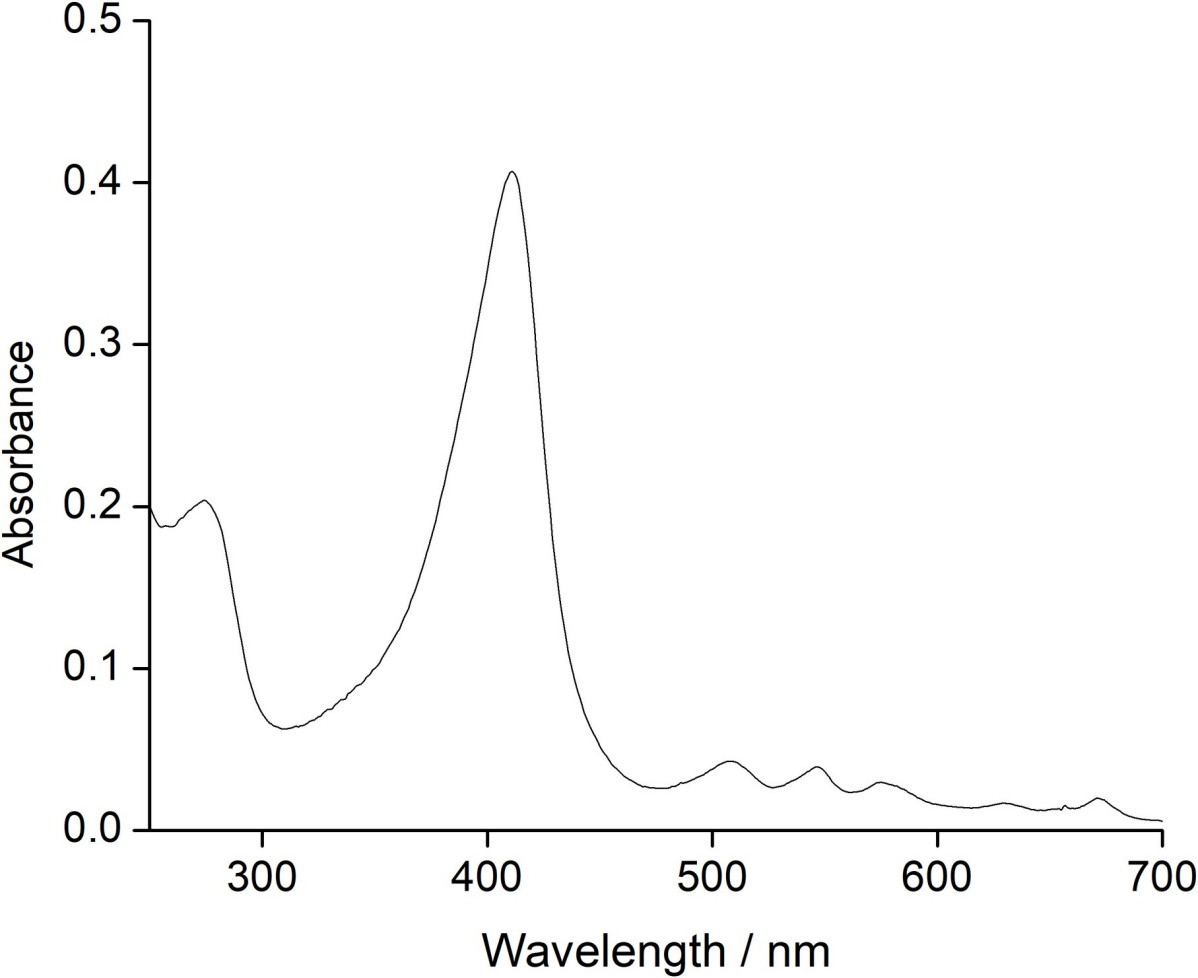
The UV–Vis spectra of PPIX reconstituted-C14A/M69A mutant after passing through desalting column. The Soret band is shifted to 410 nm due to absence of the axial ligand whereas the Q bands appear at 508, 547, 575 and 630 nm assignable to metal-free porphyrin.

After the incorporation attained equilibrium, the simple PPIX reconstituted-C14A/M69F passed through a desalting column (PD 10) followed by a strong hydrophobic butyl-sepharose column, showing no removal or degradation of the PPIX, which was evident from the UV–Vis spectra after the column treatment as shown in Fig 6. This observation indicates very tight binding of the simple PPIX in the C14A/M69F double mutant inside its scaffold even in the absence of the axial ligand and coordination with the metal.

### 2.7. Thermally tolerant behavior of apo-C14A/M69F mutant before and after reconstitution

**Fig 7:**
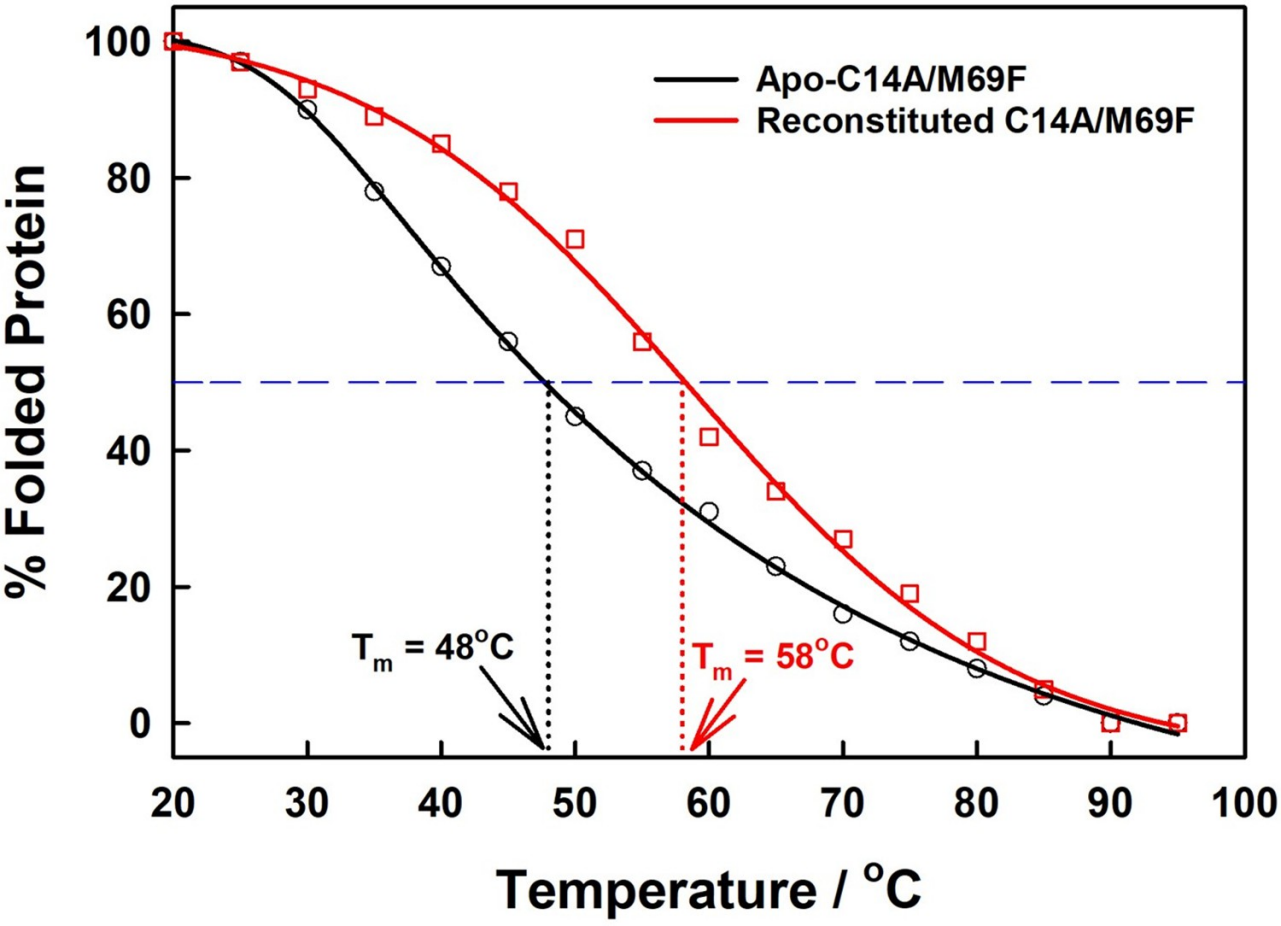
Comparison of thermo-stability of C14A/M69F before and after the reconstitution monitored by the temperature dependence of the CD spectra at 222 nm in the presence of 2M Guanidine•HCl. The black line corresponds to the apo-C14A/M69F double mutant and the red line corresponds to the apo-C14A/M69F mutant reconstituted with simple PPIX without the metal.

As in the case of apo-C14A/M69A double mutant, apo-C14A/M69F mutant also shows no visible denaturation in simple buffer solution, indicating its high thermally resistance behavior as that of the WT variant. However, in the presence of strong denaturation agent 2 M guanidine·HCl, apparent melting was detected with the T_m_ (melting point) value observed at 48°C, which is still better than the C14A/M69A variant (Fig 7). Upon reconstitution of apo-C14A/M69F with simple PPIX, sharp enhancement in the melting point is also observed in 2 M guanidine·HCl, where the T_m_ was found to be 58°C (Fig 7), which is close to the WT as well as our previous reconstituted C14A single mutant reported earlier [39–41]. All the T_m_ values in the CD spectra are calculated by the identification of the midpoint in the CD spectra, where equal fractions of folded and unfolded protein are present, resulting in the equilibrium as reported in our previous reports [40,41].

## 3. Discussion

### 3.1. An axial ligand-less incorporation of heme into apo-C14A scaffold

It was previously established that the apo-C14A scaffold retained all of its tertiary structure upon the removal of the prosthetic group and contains a vacant site in the place of its natural prosthetic group that would be able to accommodate an artificial prosthetic group [40]. In addition to these, the apo-form was also found to be thermally stable. Therefore, in order to establish a higher versatility of the scaffold and to evaluate the importance of axial ligand for the reconstitution process, metal-free porphyrin is inserted into the apo-C14A scaffold, where it shows a slow but full and quantitative reconstitution and required more than an hour to be fully incorporated. This is rather slow compared with the C14A mutant with other metalloporphyrins, where it was accomplished within a few minutes. This concludes the fact that the axial ligand is important as the reconstitution process is driven by the coordination with the metal but is not essential in the reconstitution process.

### 3.2. Properties of C14A/M69A and C14A/M69F double mutant

**Fig 8:**
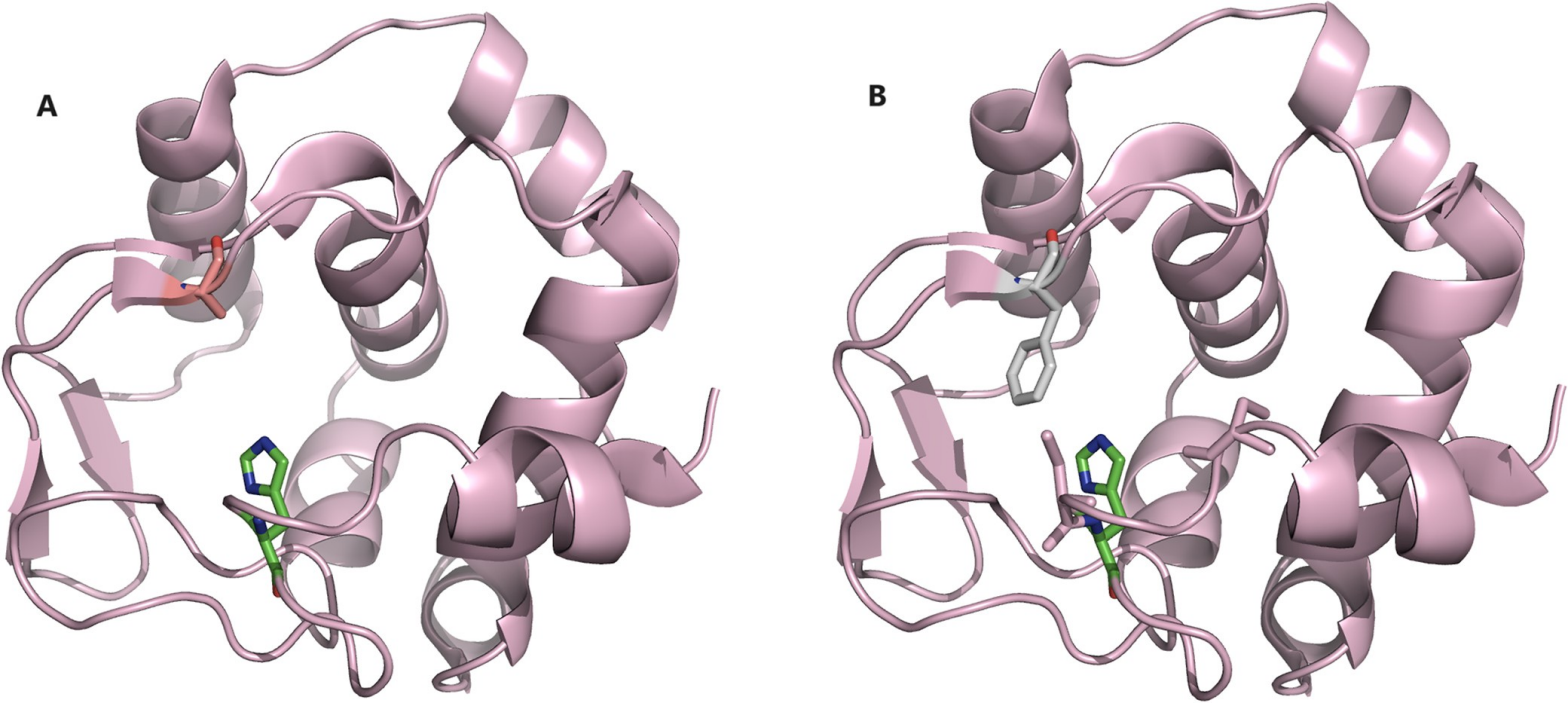
Plausible structure of A. C14A/M69A and B. C14A/M69F double mutants with the retention of His15 axial ligand and mutation in the Met69 residue. The molecular models were produced by PyMol (Schrodinger, Inc.) using the PDB file 1QYZ.

In order to further establish that the axial ligand is not crucial for the reconstitution process in the C14A scaffold, a C14A/M69A mutant was prepared in which an axial ligand in the distal side (Met69) was removed to introduce a non-coordinating alanine residue. Despite the lack of the axial ligand, the C14A/M69A mutant was successfully expressed in *E*. *coli* cells, although the protein was yielded as a mixture of holo- and apo-forms. The apo- and holo-forms of the C14A/M69A mutant were inseparable by SEC, suggesting that the apparent volumes of the apo- and holo-C14A/M69A mutants are comparable to C14A. A further CD spectrum suggests a very stable secondary structure of the apo-C14A/M69A mutant, indicating the possibility to retain most of its folded structure even after the removal of the axial ligand and native prosthetic group; it could therefore be competitive to accommodate an artificial prosthetic group with a comparable size to that of the natural heme in its vacant active site. Reconstitution of apo-C14A/M69A mutant was successful with ferric heme *b*, with the full recovery of the five coordinated heme species firmly establishing that the sixth axial ligand is not essential in the reconstitution process. A similar trend is also observed for C14A/M69F mutant as evidenced by the CD spectra and column treatment, indicating near retention of its tertiary structure after the removal of the natural prosthetic group in its apo-form (Fig 8).

### 3.3. The unusual thermal stability of C14A/M69F mutant

On the basis of the findings from the SEC column that the C14A/M69F mutant retained its full structural conformity, detailed CD spectrometer studies were carried out to investigate its thermal stability. A temperature-dependent CD spectrum of the apo-C14A/M69F shows no significant denaturation in succinic acid buffer solution until the boiling point of water. However, in the presence of strong denaturing agent 2M guanidin.HCl solution, partial conformational loss was observed with the melting point detected at 48°C. Interestingly, the loss of structure was found to be fully reversible, i.e., the conformation was found to be fully recoverable upon dialysis and subsequent removal of the guanidin.HCl from the protein solution. In fact, a full recovery of the negative cotton effect in the CD spectra was observed upon cooling the protein solution from 95°C back to normal room temperature at 30°C. The reappearance of the band at 222 nm indicates that the protein regained its secondary structure upon successive dialysis, with CD spectra after dialysis looking identical to that of the protein before Gu.HCl treatment. This investigation concludes an unprecedented thermal stability for C14A/M69F double mutant, even in the presence of a denaturing agent and elevated temperature. A more detailed study of the metal-free porphyrin reconstituted C14A/M69F double mutant also proved to be highly thermally tolerant compared to that of the WT variant, where it shows an apparent melting point at 58°C in the presence of 2M guanidin.HCl. This is an excellent match with the WT variant, indicating that even the removal of the distant axial ligand from the native heme group does not significantly alter the thermal stability of the protein scaffold. A rational explanation can be given by the fact that the reconstituted porphyrin ring is stabilized by the hydrophobic π–interactions such as π-σ/ π-alkyl with the phenyl ring mutated into the 69^th^ position of the polypeptide chain with close proximity (Fig 8B). This model concludes that the absence of axial ligand can be overcome through π-interactions if placed in a proper position.

### 3.4. Metal-free porphyrin is stabilized by various interactions inside the protein scaffold

**Fig 9:**
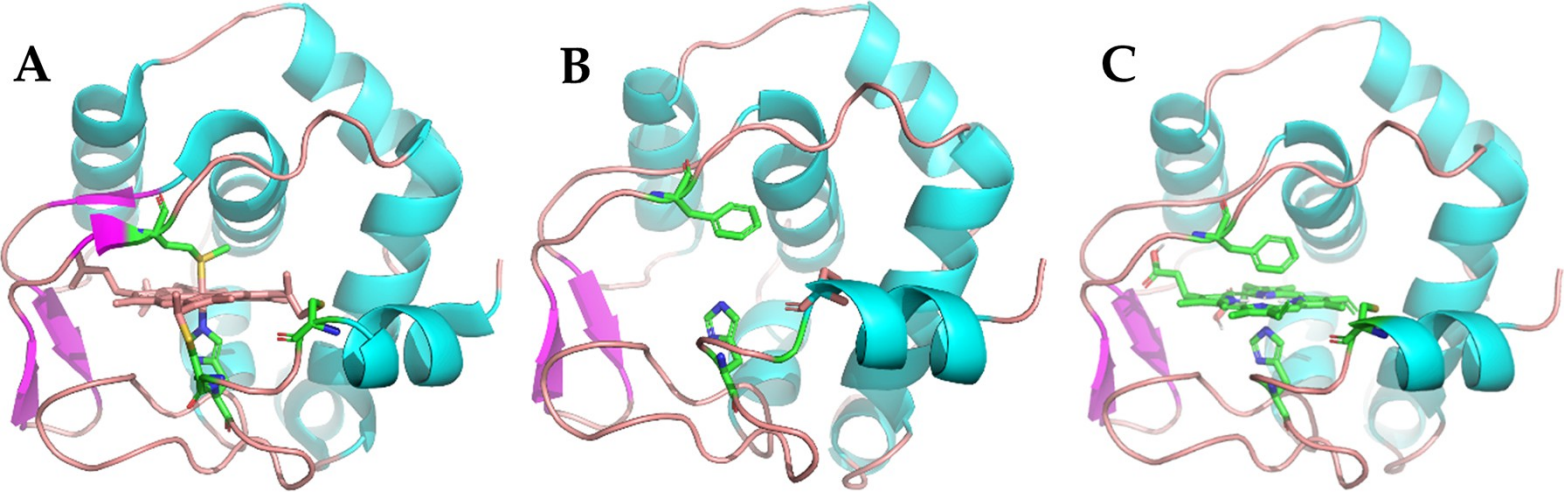
Comparison of representative structures of Cytochrome proteins. (A) Crystal structure of Cytochrome *c*_552_ protein with native heme species (PDB entry 1QYZ). (B). Plausible structure of apo-C14A/M69F engineered protein after removing the native heme species. (C**)** Docking simulation of metal-free porphyrin in the apo-C14A/M69F engineered protein. The molecular models of engineered proteins were created by PyMol (Schrodinger, Inc.) through mutagenesis using the PDB file 1QYZ.

Molecular docking studies were performed in order to rationalize all the interactions after the reconstitution of the metal-free porphyrin into apo-C14A/M69 scaffold. The results conclude that the metal-free porphyrin is docked into the same pocket of the protein scaffold that earlier accommodated the native heme species before its removal and produce an excellent superimposable structure as shown in Fig 9.

**Fig 10:**
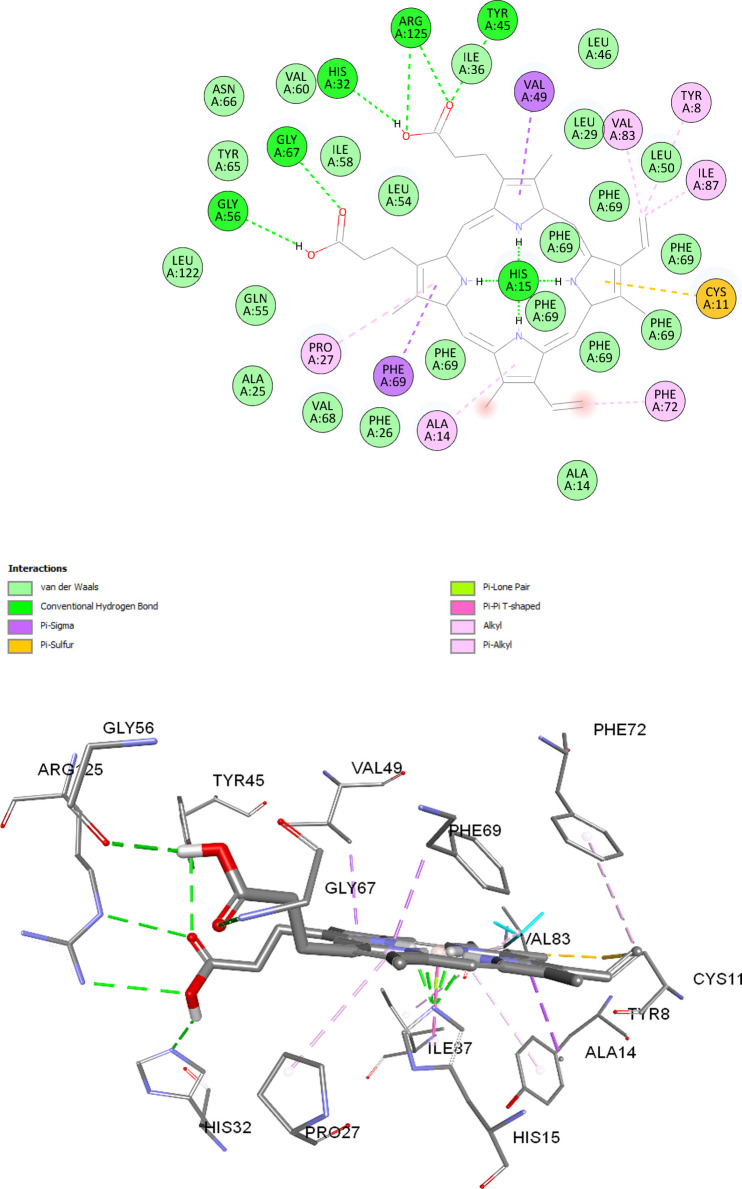
A (top). Two-dimensional diagram. B (bottom). Three-dimensional diagram for C14A/M69F-porphyrin interactions inside the heme-pocket of the engineered C14A/M69F protein scaffold.

The details of the molecular docking results are given in Table 1 and the respective 2D and 3D interaction diagrams are shown in Fig 10A and 10B, respectively.

**Table 1:** Docking interactions of the metal-free porphyrin with the engineered C14A/M69F protein inside the heme pocket.

Category	Type	Residue	Distance (Å)
Hydrogen Bond	Conventional	TYR45	3.09
Hydrogen Bond	Conventional	GLY67	3.10
Hydrogen Bond	Conventional	ARG125	3.10
Hydrogen Bond	Conventional	ARG125	3.27
Hydrogen Bond	Conventional	HIS15	2.12
Hydrogen Bond	Conventional	HIS15	2.23
Hydrogen Bond	Conventional	HIS15	2.16
Hydrogen Bond	Conventional	HIS15	2.19
Hydrogen Bond	Conventional	HIS32	2.13
Hydrogen Bond	Conventional	GLY56	2.26
Hydrophobic	Pi-Sigma	ALA14	3.15
Hydrophobic	Pi-Sigma	PHE69	3.63
Hydrophobic	Pi-Sigma	VAL49	3.66
Other	Pi-Sulfur	CYS11	5.32
Other	Pi-Lone Pair	HIS15	1.92
Hydrophobic	Pi-Pi T-shaped	HIS15	3.07
Hydrophobic	Alkyl	VAL83	4.23
Hydrophobic	Alkyl	ILE87	3.92
Hydrophobic	Pi-Alkyl	TYR8	5.07
Hydrophobic	Pi-Alkyl	PRO27	4.71
Hydrophobic	Pi-Alkyl	PHE72	5.37

The two carboxylic acid groups of the metal-free porphyrin are engaged with several strong H-bonding interactions with amino acid residues, including Gly56, Gly67, Arg125, His32, and Tyr45. Additionally, there are four hydrogen bonds between His15 and pyrrole hydrogens. A π-lone pair and π- π T-shaped interactions can also be observed engaging His15. These give the initial stabilization in the pocket during the incorporation process and indicates the necessities of His15 in the reconstitution process. Additionally, the free cysteine (Cys11), which is present in the engineered protein, interacted with the porphyrin ring through π–sulfur interaction, resulting in further stabilization. Finally, the phenylalanine residue that replaced Met69 residue in the engineered protein in (Phe69) underwent several interactions, not only though various weak Van der Waals interactions, but also through strong hydrophobic π–alkyl and/or π–σ interactions. Incidentally, these interactions with Phe69 were absent in the case of the previous C14A mutant with metal-free porphyrin and the C14A/M69A double mutant with ferric heme *b* causing their relatively higher destabilization compared with the C14A/M69F double mutant, evident by the UV–Vis spectra and CD spectra data. A π–alkyl interaction can also be observed between the vinyl group of metal-free porphyrin with the Phe72 residue that is present adjacent to the Phe69 residues as well as with Tyr8 and Pro27 residues of the polypeptide chain.

## 4. Materials and methods

### 4.1. Chemicals

All chemicals were purchased from Nakarai Tesque, Wako Co. (Tokyo, Japan), and Sigma-Aldrich (Tokyo, Japan) and used without further purification.

### 4.2. Expression of rC_552_ C14A mutant, C14A/M69A and C14A/M69F double mutants in E. coli

Constructions of the expression system for *rC*_552_ C14A mutant were performed as per our previous reports [40]. The C14A/M69A double mutant was constructed using a Quick Change Site-Directed Mutagenesis Kit (Stratagene Co.) using the expression plasmid for C14A as a template. The oligonucleotide 5′-GAAGTACAACGGCGTCGCGTCCTCCTTCGC-3′ and its complementary strand were used as mutation primers for the replacement of Met69 with Ala. The underlined sequence corresponds to the amino acid residue of mutagenesis. A bacterial strain, *E*. *coli* BL21(DE3), was used as a host for overproduction of the *rC*_552_ C14A/M69A double mutant.

A culture medium (150 mL LB added 50 μg/mL ampicilline) was inoculated from a plate of BL21(DE3) cells containing pET-*rC*_552_ C14A/M69A. After incubation for 18 h at 30°C, the culture was used to inoculate 7 L of an LB medium. This culture was incubated for 18 h with shaking at 37°C in a jar fermenter at 180 rpm. No IPTG was added for induction. The cells were harvested by centrifugation at 7000g for 10 min and stored at −80°C until use.

Constructions of the expression system for C14A/M69F double mutant were performed with a similar process to that shown for C14A/M69A double mutant through the Quick Change Site-Directed Mutagenesis Kit (Stratagene Co.) using the expression plasmid for C14A as a template with the only difference being in the primers, where 5′-GAAGTACAACGGCGTCTTCTCCTCCTTCGC-3′ is used as a primer for the replacement of the Met69 along with its complementary strand (underlined sequence correspond to the amino acid residue of mutagenesis). The subsequent inoculation, culture and harvesting were followed exactly with the same procedure as C14A/M69A double mutant mentioned above.

### 4.3. Purification of rC_552_ C14A, C14A/M69A and C14A/M69F double mutants

Purification of *rC*_552_ C14A mutant was performed according to our previously reported method [40]. Purification of C14A/M69A double mutant was performed according to the same procedure as C14A with slight modification. The whole purification process was carried out at 4°C unless specified. Frozen *E*. *coli* pellets (15 g) were thawed in ice and resuspended in 60 mL of 30 mM succinic acid–NaOH buffer, pH 5.0 (succinic buffer), and sonicated. The suspension was then centrifuged at 17500 rpm for 45 min, and the resultant supernatant was loaded into a pre-equilibrated CM-Sepharose column (120 mL) with succinic buffer and eluted with a linear gradient of 1 M NaCl of the same buffer. Reddish-green colored fractions were collected and concentrated by ultrafiltration to 1 mL along with the buffer exchange to succinic buffer. The concentrated solution was passed through a Q-Sepharose column (5 mL) pre-equilibrated with succinic buffer to remove residual nucleotides and impurities. An additional CM-Sepharose column (100 mL) pre-equilibrated with succinic buffer was applied after the Q-Sepharose column operation for final purification, which is a mixture of 5 coordinated holo *rC*_552_ C14A/M69A and apo-C14A/M69A mutant.

The resultant aqueous solution was added (NH_4_)_2_SO_4_ to achieve a final concentration of 2 M and then loaded on a pre-equilibrated butyl-Sepharose column (70 mL) with the succinic acid buffer containing 2 M (NH_4_)_2_SO_4_ (pH 5.0). The apo-C14A/M69A was eluted by a linear gradient against the succinic acid buffer. Fractions containing apo-C14A/M69A were identified by values higher than 7.0 in the ratio of absorbance at 280 over 420 nm as reported previously and were used for further investigations [40].

Purification of *rC*_552_ C14A/M69F double mutant was performed according to the same procedure as C14A/M69A double mutant, resulting in a mixture of apo- and holoproteins. Subsequent separation of holo and apo-C14A/M69A double mutant was performed by passing through a butyl-Sepharose column.

### 4.4. Reconstitution of apo-C14A, apo-C14A/M69A and apo-C14A/M69F mutants

Reconstitution of apo-C14A/M69A was achieved by titration of apo-C14A/M69A against ferric heme *b* at 25°C in succinic acid buffer. A measure of 5 mM stock solution of Fe(III)-PPIX was prepared by dissolving it in 0.01 M NaOH, which was then added to succinic buffer to make the final concentration of 5 μM. Then, they were titrated against apo-C14A/M69A until the final concentration of the apo-protein reached 2.5 times the concentration of hemin. The UV–Vis spectra were taken after 5 minutes of each addition. An excess amount of heme was removed from the protein using a Q-sepharose column.

Reconstitution of apo-C14A and apo-C14A/M69F was achieved through mixing an equimolar amount of simple-PPIX without the metal at 25°C with the final concentration of 5μM and 8 μM, respectively. It was subjected to a time-dependent study performed during every 5 min interval in the UV–Vis spectra to achieve saturation in the Soret band and continued for 3 hrs.

### 4.5. Spectroscopy

A Shimadzu UV-2400 PC spectrometer was used to record all UV–Vis spectra. A MultiSpec-1500 spectrometer from Shimadzu Co. (Kyoto, Japan) equipped with a temperature controller was used to find heme degradation spectra in the reconstituted double mutants. Circular dichroism spectra were recorded on a J-720WN CD spectrometer from JASCO Co. (Tokyo, Japan) with a temperature controller. A thin optical cell of 1 mm path length, containing 10 μM protein solution in succinic buffer, was used for all the measurements. The temperature-dependent CD spectra were measured by raising the temperature linearly from 20 to 95°C at a rate of 1°C min^–1^ recording the 222 nm band, which corresponds to the absorption maximum of a negative Cotton effect resulting from α-helices.

### 4.6. Molecular docking

Both protein and ligand molecules were prepared for docking using Autodock Tools v1.5.6 [46] following a standard protocol [47,48]. For this, water-molecule-free protein was prepared. Subsequently, polar hydrogens were added and nonpolar ones were merged for both ligand and protein molecules followed by the addition of Kollman and Gasteiger charges to protein and ligand molecules, respectively. The generated PDBQT ensured that the protein molecules would be treated as rigid while the flexibility of the ligand was complemented by setting all bonds as rotatable. The molecular docking was then achieved by means of Autodock Vina v1.2.3 using a hybrid scoring function comprising combined knowledge-based and empirical functions [49,50]. For the docking process, a grid box with dimensions of 20 × 20 × 20 Å^3^ and a spacing of 0.375 Å was centered on the active binding site of the protein with center coordinates (x = 23.1763, y = 23.8836 and z = 26.6197). The resulting docked poses were automatically arranged by descending order of protein-ligand binding affinities. The best conformation (i.e., the lowest energy pose) out of the generated ligand poses was visualized using BIOVIA Discovery Studio Visualizer 2021 [51] and the identified receptor–ligand 3D interactions graphs were generated.

## 5. Conclusion

The present manuscript proposed a versatile Cytochrome c scaffold that can accommodate an artificial prosthetic group in its core, even in the absence of a metal. Contrary to the conventional belief that the reconstitution process is facilitated through coordination with the central metal ion in the artificial heme group, in the present study, we successfully reconstituted a metal-free protoporphyrin inside the thermally tolerant apo-scaffold to yield a new type of engineered protein where none of the axial–metal coordination bonds are present. A detailed molecular docking investigation was performed, which explained the stability of the metal-free porphyrin in the engineered protein scaffold through various kinds of interactions such as hydrogen bonding and hydrophobic interactions in addition to hydrophobic π–alkyl and/or π–σ interaction with the phenylalanine residue at the 69^th^ position of engineered protein and the inserted ligand in its pocket. Significant stabilization was also done by His15 which is interacting with the metal-free porphyrin through π- π T-Shaped and π-lone pair interactions. This achievement opens a wider choice for the metal-free artificial prosthetic group and other chromophores, which can be used as active sites in the engineered cytochrome proteins. The present engineered proteins are also highly thermally tolerant under severe conditions such as the addition of an external denaturating agent. A complementary biophysical study of the mutation effect on protein stability (protein folding thermodynamics) along with kinetics investigations of the reconstitution process would shed more light on the presented findings. This will be hopefully the subject of our future work which is expected to lead to a better understanding of the unusual thermal stability. Proper reconstitution with a metal-bearing artificial heme species resulting in a 5-coordinated active site in the engineered protein can also provide additional advantages for constructing a novel artificial enzyme or biocatalyst, as the vacant space in the distant axial ligand could be pivotal for the incorporation of the substrate in the catalytic reaction upon rational engineering.

## Supporting information

S1 File Supplementary material. (PDF)
