# Supplementary material for: An engineered thermally tolerant apo-cytochrome scaffold for metal-less incorporation of heme derivative
Source: PLoS One. 2023 Nov 9;18(11):e0293972. doi: 10.1371/journal.pone.0293972 (PMC10635480; doi:10.1371/journal.pone.0293972)
Supplement: S1 File — (PDF) [file pone.0293972.s001.pdf]

Supplementary material

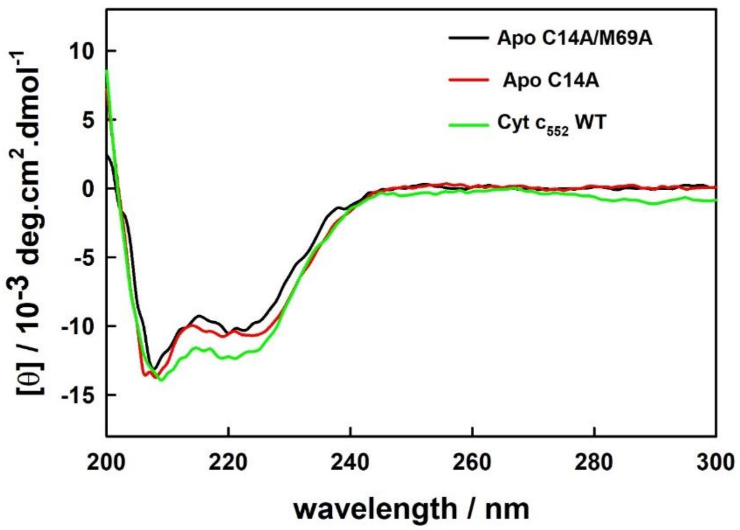

Fig S1. CD spectroscopy of apo-C14A/M69A double mutant in comparison with C14A and WT. The black line corresponding to apo-C14A/M69A double mutant whereas red and green line corresponding to apo- rC_{552} C14A and WT respectively.

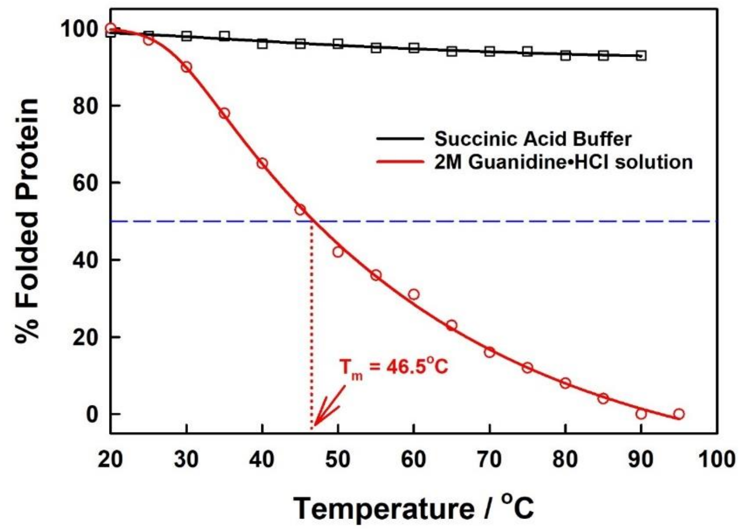

Fig S2. Comparison of thermo-stability of reconstituted apo-C14A/M69A double mutant monitored by the temperature dependence of the CD spectra at 222 nm. The black line corresponding to the percentage of folded structure in presence of succinic acid buffer whereas the red line corresponding to the folded structure in presence of 2M Guanidine•HCl.
